# Vitamin D and multiple sclerosis

**Published:** 2014

**Authors:** Asghar Amini Harandi, Ali Amini Harandi, Hossein Pakdaman, Mohammad Ali Sahraian

**Affiliations:** 1 Department of Biochemistry, School of Medicine, Jahrom University of Medical Sciences, Jahrom, Iran; 2 Department of Neurology, School of Medicine, Shahid Beheshti University of Medical Sciences, Tehran, Iran; 3 MS Research Center, Neuroscience Institute, Tehran University of Medical Sciences, Tehran, Iran

**Keywords:** Multiple Sclerosis, Vitamin D, Vitamin D Receptor, Polymorphism

## Abstract

Multiple sclerosis (MS) is a chronic demyelinating disease and also is one of the most common disabling neurological disorders in young and middle-aged adults. The main pathogenesis of MS has long been thought to be an immune mediated disorder of the central nervous system. The function of the immune system is under the influence of vitamin D which as a modulator of immune response could play a role in autoimmune diseases including MS. Deficiency of vitamin D or variations in DNA sequence (polymorphism) of vitamin D receptor gene diminishes its optimal function on immune system that consequently could lead to increasing risk of MS. However, its role in development and modulating the course of MS is still under investigation. In this review we aimed to discuss the role of vitamin D in body, immune system and consequently altering the risk of MS.

## Introduction

MS is an immune mediated disorder of the central nervous system, in which T-cells and other immune effector populations entering the brain and attack the nerve cells, strip away their myelin insulation and destroy their axons and entire remaining structures.^1, 2^ MS is thought to affect more than 2.1 million people worldwide and its prevalence is increasing in the most region in the world.^3^ Pathogenesis of MS is dependent on both genetic and environmental susceptibility factors^3^, and is widely believed to have a T-cell-mediated autoimmune etiology.^4^ Strong evidences from genetic epidemiologic studies also imply that environmental factors affect susceptibility to MS at a broad population level.^5^ Even exposure to the same environment, individual susceptibility to MS may be different, which indicates that genetic factors are important in the pathogenesis of MS. Linkage and association studies have found that the human leukocyte antigen (HLA) regions are the strongest susceptibility locus for MS, but it does not completely explain the genetic impact on disease susceptibility.^6^ Conversely, recent studies of genome scans suggest that no single MS susceptibility locus is present or sufficient to cause the disease.^7^ In fact, MS is a heterogeneous disorder, so that different genes might influence the course or the presentation of the disease.^8^

Also genes lying in non-HLA regions may also alter susceptibility for MS. In the past decade, considerable efforts and expense were put into attempts to detect the non-HLA polymorphisms contributing to the risk for MS.^9^

Many other immunopathological processes such as dysfunction of regulatory T cells, inflammation, and primary apoptosis of oligo-dendrocytes or B cell mediated autoimmunity have been demonstrated to be involved in MS pathogenesis. Finally Vitamin D is a potent modulator of immune system therefore vitamin D deficiency is the most prominent candidate for MS and is focus of interest in field of MS during recent years.

### Vitamin D as an immune modulator

Vitamin D is a steroid hormone that has multiple regulatory and functional effects throughout the body. Vitamin D endocrine system has a central role in control of bone and calcium homeostasis. It can be obtained either through dietary intake and supplements or produced endogenously. It found in foods such as oily fish, egg yolks, fortified milk and juice.^10^ However dietary intake accounts only for about 30% of the vitamin D obtained and is not biologically active^11^, therefore the main route for obtaining vitamin D is exposure to ultraviolet B sunlight at wavelengths between 290–315 nm that occur predominantly in the summer months.^12^

The biologically active form of vitamin D is 1,25-dihydroxyvitamin D3 (1,25(OH)2D3) that is produced in several steps. The most of vitamin D is obtained by skin in a UVB-induced process in which 7-dehydrocholesterol convert to vitamin D3 followed by two hydroxylations. First, in the liver, and then in the kidneys by 25 and 1 hydroxylases, respectively. Finally active metabolite can enter the cell, bind to the vitamin D-receptor and subsequently to a responsive gene.^13^

A central role of 1,25(OH)2D3 is modulation of immune response.^14^ This is suggested by the fact that many immune cells including monocytes, macrophages, dendritic cells and activated T and B cells contain vitamin D receptors. It control infections by promoting differentiation of monocytes to macrophages^15^, and enhance their chemotactic and phagocytotic capacity and antibacterial activity .^16^ It also has a preventive role against autoimmunity by reducing the expression of major histocompatibility complex (MHC) class II and co-stimulatory receptors on antigen presenting cells. It acts as a co-stimulatory molecules on monocytes and T cells.^17, 18^ Furthermore, 1,25-(OH)2D3 induces proliferation of B cells, differentiation of plasma cells, secretion of immunoglobulin E and M, production of memory B cells, and apoptosis in activated B cells.^19^ In addition promotes T regulatory cells and reduces T cell proliferation, inhibits production of pro-inflammatory cytokines. Overall, existing data indicate that 1,25-(OH)2D3 modulates several players of the immune cascade to generate a more anti-inflammatory and tolerogenic profile.

It has been cleared that treatment by 1,25(OH)2D3 suppress the development of Th1-mediated autoimmune diseases.^20–23^ In addition treatment of mice with MS symptoms by 1,25(OH)2D3, interrupt the development of the disease in these mice. Such findings may explain that vitamin D can change the immune response even after the disease had been established.^20^

T cells and ultimately B cells, both may be subject as a direct or indirect targets of 1,25-(OH)2D3. Increasing of vitamin D receptor expression after T cell activation support this hypothesis that T cells are main targets of 1,25-(OH)2D3. However, different activation stimuli appear to evoke diverse vitamin D receptor (VDR) expression levels and kinetics, which may explain the inconsistency in data concerning the effects of 1,25-(OH)2D3 on T cell proliferation.^24^

In addition, 1,25(OH)2D up-regulate many genes such as osteocalcin, osteopontin, calbindin, and 24-hydroxylase.^25^ For example, vitamin D metabolites may also have a protective role against diabetes mellitus type 1 by down-regulation of dendritic and Th1 cells, suppression of the antigen-presenting capacity of macrophages and dendritic cells and promotion of Th2 lymphocytes.^26^

More recent findings have also linked vitamin D deficiency to a range of non-skeletal conditions such as cardiovascular disease, cancer, stroke, Cognitive impairment and dementia. 1,25(OH)2D has shown neuro-protective effects including the clearance of amyloid plaques, a hallmark of Alzheimer's Disease. An association has been noted between low 25-hydroxyvitamin D [25(OH)D] and Alzheimer's disease and dementia in both Europe and the United States.^27^

### Vitamin D and auto-immune disease

The role of vitamin D has been investigated in several autoimmune diseases, including inflammatory bowel disease (IBD), autoimmune thyroiditis, rheumatoid arthritis (RA), type 1 diabetes mellitus, mixed connective tissue disease, scleroderma, systemic lupus erythematosus (SLE), allergic encephalomyelitis and MS.^28–33^ It prevents development of autoimmune diseases in animal models.^34, 35^ Some studies suggest that risk of some disease such as rheumatoid arthritis, and diabetes mellitus type I, reduce in human with high vitamin D intake.^36, 37^

The best indicator of vitamin D status, reflecting intake from all sources, is serum 1,25-(OH)2D3. Serum levels of less than 25 nmol/L considered as severely deficient, levels between 25-80 nmol/L considered as insufficient of mildly to moderately deficient and levels more than 80 nmol/L considered as sufficient.

Patients with MS have low serum levels of vitamin D compared to the international norm, and it has been hypothesized vitamin D plays an immune-modulatory role in the CNS, perhaps through a Th1-mediated response.^38^

Vitamin D is an in vitro potent immune modulator, that can ameliorate, or even cure, animal models with MS.^28^ In the other word, a poor vitamin D status could increase risk for MS^39, 40^ and lead to a more severe disease course of MS.^41^ Several experimental studies, have shown that vitamin D brings the immune system in a less pro-inflammatory state.^28^ This important role of vitamin D in preventing immune deviation has been argued to underlie the association of a poor vitamin D status with MS. However, the causality of this association is not thoroughly clear.^42^

### Vitamin D signaling is mediated by binding of 1,25(OH)2D3 to the VDR

VDR is an intracellular receptor belonging to the steroid/thyroid nuclear receptor family, that has been found in the brain, on immune cells, peripheral blood monocytes as well as multiple other tissues.^43, 44^ The presence of the VDR in both the thymus and the peripheral T cells indicate critical role for vitamin D in both development and function of T cells. VDR form a heterodimer with the retinoid X receptor. After translocation to the nucleus, this heterodimer in complex with transcription factor IIB binds to a vitamin D response elements (VDREs) in target genes involved in various processes including cell proliferation, differentiation, and immunomodulation.^45^ Binding of 1,25(OH)2D to VDR/RXR stabilizes the heterodimer, resulting in an increased amount of heterodimers in the nucleus and an increased transactivation or trans repression of target genes.^46^

Vitamin D3 by bonding to VDR inhibits the production of the interleukins (IL) 1, 2, 6 and 12, interferon (IFN) γ, and tumor necrosis factors (TNF) α and β.^47^ These cytokines play effective roles in the development of T helper (Th) 1 cells, which are involved in the pathogenesis of chronic inflammatory autoimmune diseases.^48^ Some studies show that administration of 1,25(OH)2D3 completely prevent experimental autoimmune encephalomyelitis (EAE), that is one of the most useful models of MS.^20^

Interestingly 1,25(OH)2D3 increase development of Th2 cells via a direct effect on naive CD4+ cells^49^ and to induce regulatory T cells.^50^ Consequently, VDR agonists appear to primarily inhibit pro inflammatory, pathogenic T cells such as Th1 and Th17 cells, and to favor development of Th2 or T regulatory cells.^51^ The anti-inflammatory, immune regulatory and pro-tolerogenic properties of VDR agonists indicate their important role in the physiological regulation of innate and adaptive immune responses and suggest their development as potential therapies for autoimmune disorders.^51, 52^

### VDR polymorphisms as a candidate for auto-immune diseases

Most of the biological activities of 1,25(OH)2D3 are mediated by Vitamin D receptor that acts as a ligand-activated transcription factor.^53^ VDR agonists have recently been identified as potent immune modulators capable of inhibiting Th1-mediated immune response. Consequently, genetic alterations of the VDR gene could lead to important defects on gene activation, affecting calcium metabolism, cell proliferation and might contribute to increase risk for developing autoimmune diseases.^54^ Variations in sequence of DNA, which occur frequently in the population, are referred as “polymorphisms” and can have modest and subtle but true biological effects. Their abundance in the human genome and its high frequencies in the human population have made them targets to explain variation as a risk of common diseases.^55^

The gene encoding for VDR is located on chromosome 12q13.1, and contains near 100 kb that divided in 8 introns and 9 exons. The first exon is located in the promoter region of the gene, which can generate multiple tissue specific transcripts.^55^ Exon 2 and 3 code for the DNA binding domain, and exon 6–9 for the ligand binding domain.^56^ From over 30 polymorphisms within the VDR gene that are listed by Entrez SNPs (single nucleotide polymorphisms) database of the National Center for Biotechnology Information, only a few of them has been studied in relation to several autoimmune diseases, and even a more limited number has been related to immune regulation and MS.^57^ Investigation of VDR gene polymorphisms on the interaction between vitamin D and the immune system is an interesting field for recent studies.

The association of VDR gene polymorphisms with MS has been investigated in case-control and transmission studies, as well. The first studies which reported an association were performed in Japanese population.^49, 50^ These findings were extended in an Australian population.^58^

### Risk factors for vitamin D deficiency

The most common risk factors for vitamin D deficiency are low sun exposure, skin pigmentation, premature and dysmature birth, obesity, malabsorption, race, age and environmental factors.^13^ Vitamin D deficiency is much more frequent in Europe than in Asia and America. In Europe, the highest serum 25(OH)D3 levels were observed in Scandinavian countries and the lowest levels were found in Mediterranean countries that may be due to high sun exposure, a light skin and multivitamin use in northern countries while shadow-seeking behavior and a darker skin are more common in Mediterranean countries.^59^

A high prevalence of vitamin D insufficiency has been reported in Afro-Americans, because people with dark-colored skin have a reduced ability to synthesize vitamin D upon exposure to sunlight than those with light-colored skin.^54^ Also prevalence of vitamin D deficiency is high in non-western immigrants in the Netherlands^60^ and in the Middle East^61^ especially in Iran^62^, where life-style factors probably play a role.

Because of changes that occur with aging, older people with any other risk factors for vitamin D deficiency are likely to have inadequate stores of this vitamin. Elderly individuals also have a lower capacity to synthesize vitamin D on exposure to ultraviolet-B radiation and often stay indoors.^63^ People hospitalized for a long time and are not supplemented with vitamin D and people who wear concealing clothing for religious or cultural purposes may be at higher risk of vitamin D deficiency.^64^ Supplementation with cod-liver oil had a protective role in person with low summer outdoor activities.^65^ Vitamin D2 is not a suitable supplement for many reasons including absorption, differences in efficacy at raising vitamin D levels, diminished binding to proteins in blood, shorter shelf-life etc.

### Vitamin D and risk of MS

There is a strong relationship between vitamin D levels and risk of MS. Vitamin D level at baseline was a significant predictor of risk of developing definite MS. In women for each 10 nmol/L increase of serum 1,25(OH)2D level was associated with a 20% reduction in the likelihood of MS development suggesting a “protective” effect of higher 1,25(OH)2D serum levels.^40^

Correlation between sunlight exposure and MS is confirmed by several evidences such as higher childhood sunlight exposure associated with lower MS risk^66^, lower incidence of non-melanoma skin cancer in MS^67^, inverse correlation between MS prevalence and sunlight and inverse correlation between altitude as a marker of sunlight intensity and MS, so that higher sun exposure during childhood and early adolescence is associated with a reduced risk of MS.^68^ The geographical distribution of MS with low prevalence in equatorial regions and increasing prevalence with increasing latitudes in both hemispheres correlates with sun exposure/ vitamin D levels. MS is more common in areas further away from the equator where there is less sunshine, which show a relationship between vitamin D and the risk of developing MS.^69, 70^ Migrating populations seem to acquire the MS risk of the areas they move to but individuals keep risk of country of origin if move after adolescence^71, 72^, that confirm hypotheses of a long latent period in MS development.

One of factors that influence MS susceptibility is month of birth in which significantly fewer MS patients were born in late spring in compare to who were born in fall.^73^ Month-of-birth has also been correlated with latitude and presumptive vitamin D exposure, suggesting that mothers of babies deliver in spring have less sun exposure compared to mothers who deliver in fall.^74, 75^ This diminished in utero exposure to vitamin D, coupled to the solar cycle and latitudinal differences, may be an environmental risk factor for the development of MS.^39, 76^

Vitamin D deficiency is common in patients with MS.^77^ The cause of low vitamin D levels in patients with MS is likely due to a combination of low vitamin intakes and decreased outdoor activities in climates that are against for vitamin D synthesis in the skin.^78^

Some recent research studies suggest that a lack of vitamin D in early childhood or before birth might increase the risk of developing MS later in life. Outdoor activities during summer in early life (ages 16–20 years) were associated with a decreased risk of MS. Epidemiological study showed that women with the highest supplements of vitamin D intakes had a 40% reduction in the risk of developing MS comparing women with intake of >400 IU/day to those with no supplemental vitamin D. A dose of 1000 to 4000 IUs daily to achieve a serum level of > 99nmol/L is safe and may reduce risk by as much as 62% MS.^76^

## Conclusion

The maintenance of adequate vitamin D levels has important influence on different aspects of health and well- being. Vitamin D deficiency is a modifiable risk factor for MS and because of its role in control of immune responses almost certainly has some beneficial effects on disease course in MS.

Several studies revealed prominent role of Vitamin D in immune mediated diseases like MS. However, the exact role is not completely understood in basic and clinic. Polymorphisms of VDR may be considered as missing link that could illustrate why different patients response to Vitamin D differently, in clinical practice. However, administration of Vitamin D is increasing and become more acceptable in routine practice of patients with MS in current years.
